# AGA Clinical Practice Update on Diagnosis and Management of Acute Hepatic Porphyrias: Expert Review

**DOI:** 10.1053/j.gastro.2022.11.034

**Published:** 2023-01-13

**Authors:** Bruce Wang, Herbert L. Bonkovsky, Joseph K. Lim, Manisha Balwani

**Affiliations:** 1 Department of Medicine and Division of Gastroenterology, University of California San Francisco, San Francisco, California; 2 Section of Gastroenterology and Hepatology, Wake Forest University School of Medicine, Winston-Salem, North Carolina; 3 Section of Digestive Diseases and Yale Liver Center, Yale University School of Medicine, New Haven, Connecticut; 4 Departments of Genetics and Genomic Sciences, Icahn School of Medicine at Mount Sinai, New York, New York

**Keywords:** 5-Aminolevulinic Acid, 5-Aminolevulinic Acid Synthase, Heme, Porphobilinogen, Porphyria, Porphyrins

## Abstract

**DESCRIPTION:**

The acute hepatic porphyrias (AHP) are rare, inborn errors of heme-metabolism and include acute intermittent porphyria, hereditary coproporphyria, variegate porphyria, and porphyria due to severe deficiency of 5-aminolevulinic acid dehydratase. Acute intermittent porphyria is the most common type of AHP, with an estimated prevalence of patients with symptoms of approximately 1 in 100,000. The major clinical presentation involves attacks of severe pain, usually abdominal and generalized, without peritoneal signs or abnormalities on cross-sectional imaging. Acute attacks occur mainly in women in their childbearing years. AHP should be considered in the evaluation of all patients, and especially women aged 15–50 years with recurrent severe abdominal pain not ascribable to common causes. The screening tests of choice include random urine porphobilinogen and *δ*-aminolevulinic acid corrected to creatinine. All patients with elevations in urinary porphobilinogen and/or *δ*-aminolevulinic acid should initially be presumed to have AHP. The cornerstones of management include discontinuation of porphyrinogenic drugs and chemicals, administration of oral or intravenous dextrose and intravenous hemin, and use of analgesics and antiemetics. Diagnosis of AHP type can be confirmed after initial treatment by genetic testing for pathogenic variants in *HMBS, CPOX, PPOX*, and *ALAD* genes. AHP is also associated with chronic symptoms and long-term risk of systemic arterial hypertension, chronic renal and liver disease, and hepatocellular carcinoma. Patients who have recurrent acute attacks (4 or more per year) should be considered for prophylactic therapy with intravenous hemin or subcutaneous givosiran. Liver transplantation is curative and reserved for patients with intractable symptoms who have failed other treatment options.

**METHODS:**

This expert review was commissioned and approved by the American Gastroenterological Association (AGA) Institute Clinical Practice Updates Committee (CPUC) and the AGA Governing Board to provide timely guidance on a topic of high clinical importance to the AGA membership, and underwent internal peer review by the CPUC and external peer review through standard procedures of *Gastroenterology*. These Best Practice Advice (BPA) statements were drawn from a review of the published literature and from expert opinion. Because systematic reviews were not performed, these BPA statements do not carry formal ratings of the quality of evidence or strength of the presented considerations.

Porphyrias are inherited disorders in the heme biosynthesis pathway. Heme is an essential molecule that carries out a wide array of functions necessary for aerobic life. It is synthesized through 8 enzymatic steps, and mutations that lead to partially defective activity in heme synthesis enzymes result in the 8 inherited porphyrias.^1,2^ Symptoms are due to the specific intermediates that accumulate before the defective enzymatic step. The acute hepatic porphyrias (AHPs) include acute intermittent porphyria (AIP), variegate porphyria (VP), hereditary coproporphyria (HCP), and 5-aminolevulinic acid dehydratase deficiency porphyria (ALAD) (Table 1). They present with acute neurovisceral symptoms due to abnormal accumulation of the porphyrin precursors *δ*-aminolevulinic acid (ALA) and porphobilinogen (PBG). Clinically, they present with severe acute abdominal pain, nausea, vomiting, constipation, muscle weakness, neuropathy, tachycardia, and hypertension. Four additional porphyrias present primarily with cutaneous symptoms and are not discussed in this review.

AIP, HCP, and VP are autosomal dominant disorders and ALAD porphyria is a very rare autosomal recessive disorder with fewer than a dozen reported cases in the world literature.^3–6^ Pathogenic variants in the hydroxymethylbilane synthase (HMBS), coproporphyrinogen oxidase, and protoporphyrinogen oxidase genes (referred to collectively as AHP pathogenic variants) result in at least a 50% reduction of the activity in the corresponding enzyme (Figure 1). Symptomatic AHPs are thought to affect approximately 1 in 100,000 patients; however, data from population-level genetic studies showed that the prevalence of pathogenic variants for AIP is between 1 in 1300 and 1 in 1785, much higher than previously believed.^7,8^ Diagnoses of AHPs are often missed, with a delay of more than 15 years from initial presentation. This suggests that the true prevalence of symptomatic AHPs may be higher. Recent advances in treatment have improved the outlook for patients with AHP. This review is designed to provide best practice advice on the diagnosis, treatment, and long-term management of patients with AHP. We have developed Best Practice Advice statements to address 12 key clinical issues.

## Best Practice Advice 1: Women aged 15–50 years with unexplained, recurrent severe abdominal pain without a clear etiology after an initial workup should be considered for screening for an AHP.

Approximately 90% of patients with symptomatic AHP are women and attacks are rare before onset of menses or after menopause in these patients. Among symptomatic patients with AHP, >90% experience only 1 or a few acute attacks in their lifetimes. Attacks are often precipitated by inducing factors that increase heme production in the liver. An estimated 3%–5% of patients with symptomatic AHP experience frequent recurrent attacks, typically defined as 4 or more attacks per year. These attacks are often not associated with identifiable triggers, although some attacks during the luteal phase of the menstrual cycles are believed to be triggered by progesterone. In addition to the acute attack symptoms described, >50% of patients who experience recurrent attacks report chronic neurologic symptoms, and 35% have received a diagnosis of neuropathy.^9,10^ These patients have a markedly impaired quality of life^11,12^ and are at higher risk of long-term complications of AHP, including hepatocellular carcinoma (HCC)^13^ and chronic renal failure.^14,15^ ALA and PBG levels in patients experiencing recurrent attacks may be elevated at baseline between attacks.^16^ It remains unclear whether chronic symptoms are due to persistently elevated ALA or incomplete recovery from neurologic injury sustained during acute attacks.

Some individuals who cany an AHP pathogenic variant have elevated ALA and PBG but have never experienced acute attacks. This group of asymptomatic high excretors may be at increased risk for an induced acute attack, chronic renal or hepatic injuiy, and/or HCC relative to mutation carriers with normal ALA.^17,18^ Diagnosis of AHPs is frequently delayed, with an average of 15 years from onset of symptomatic AHP to diagnosis in the United States and Europe.^19^ Accordingly, AHP should be considered in any patient, especially any woman of childbearing age, who presents with unexplained recurrent, severe abdominal pain.

## Best Practice Advice 2: Initial diagnosis of AHP should be made by biochemical testing measuring ALA, PBG, porphyrins, and creatinine in a random urine sample.

Diagnosis of symptomatic AHP requires biochemical testing. The hallmark of acute attacks is significantly elevated ALA and PBG in the urine or plasma. In all AHP except ALAD porphyria, PBG is elevated. During acute attacks, both ALA and PBG are elevated at least 5-fold the upper limit of normal. The levels are high enough that a random urine sample is sufficient and a 24-hour urine collection is not recommended. To adjust for differences in the degree of urinary concentration, ALA and PBG excretion should be normalized to that of creatinine. It is important to note that because ALA and PBG are porphyrin precursors, they are not included in tests of porphyrins, which typically measure porphyrins in urine or stool that have been separated and quantified by high-performance liquid chromatography with fluorescence detection. Urine porphyrins should not be used alone as a screening test for AHP. Mild and nondiagnostic elevations in urinary porphyrins (secondary porphyrinurias) are often incorrectly interpreted as indicating AHP and lead to erroneous overdiagnosis.

Both ALA and PBG can be measured with high sensitivity and specificity.^20–22^ Unfortunately, these tests are performed at large reference laboratories only, and results often require 1–2 weeks to be reported. Rapid, qualitative urine PBG tests have been available since the 1950s (Hoesch or Watson-Schwartz test),^23^ but their clinical use has been limited due to lack of use by most hospitals. Recently, a new rapid test for PBG was approved in the United States (Teco Diagnostics).

For most patients with AHP, those who experience only a few acute attacks in their lifetimes, testing for urine ALA, PBG, and creatinine is most useful during an acute attack. Unless the patient has been treated with intravenous hemin, testing for ALA and PBG can be performed days after the acute attack. Studies of patients with AIP found that ALA and PBG can remain elevated in urine for months to years after an acute attack.^24^ However, ALA and PBG levels can fall quickly after an acute attack in patients with HCP or VP. When testing is performed in patients with sporadic AIP when they are asymptomatic, 15%–44% can have normal urine ALA and PBG values.^25,26^ The same, or even higher, percentages are likely true for HCP and VP. In these patients, confirmatory testing may require repeat testing during an acute attack.

In the small population of patients with AHP with recurrent acute attacks, urine ALA and PBG are typically elevated even at baseline between acute attacks.^16^ In patients who have frequent symptoms suggestive of acute attacks, normal ALA and PBG levels likely rule out AHP as the etiology of the symptoms.

## Best Practice Advice 3: Genetic testing should be used to confirm the diagnosis of AHP in patients with positive biochemical testing.

Once the biochemical tests indicate AHP, confirmation of the specific type of AHP is usually established by genetic testing, with sequencing of the 4 genes *ALAD, HMBS, CPOX*, and *PPOX* leading to ALAD, AIP, HCP, and VP, respectively. When whole-gene sequencing is performed, 95%–99% of cases can be identified.^27^ First-degree family members should be screened with genetic testing once the familial pathogenic variant has been identified in the patient with AHP to identify patients at risk for acute attacks. Those who are mutation carriers should be counseled.

Although genetic testing is the gold standard for confirmation of diagnosis, it is not recommended for initial screening. Most carriers of pathogenic variants in AHP genes do not experience symptomatic acute attacks in their lifetimes. The estimated phenotypic penetrance of symptomatic disease is approximately 1% of AIP gene carriers,^8^ with a penetrance of >20% in families with symptomatic patients.^7^ HCP and VP are reported to have lower penetrance than AIP.^28^ Even among patients with a confirmed pathogenic variant, attribution of clinical symptoms often benefits from biochemical confirmation (Table 2).

## Best Practice Advice 4: Acute attacks of AHP that are severe enough to require hospital admission should be treated with intravenous hemin, given daily, preferably into a high-flow central vein.

The currently approved treatment for acute attacks is intravenous hemin infusion,^29–33^ usually given once daily at a dose of 3–4 mg/kg body weight, typically for 4 days. Hemin rapidly down-regulates ALAS1 expression in the liver, thus ameliorating the continued overproduction and accumulation of ALA and PBG. Symptom relief depends on elimination of excess ALA and PBG and typically requires 48–72 hours, although recovery from neurologic symptoms can vary significantly. Timely initiation of hemin therapy results in normalization of ALA and PBG levels, symptom improvement, and decreased risk of long-term neurologic complications.

A random urine for ALA, PBG, and creatinine should be collected before the start of hemin treatment. Due to the lack of available rapid ALA or PBG tests, initiation of hemin can be made on empirical grounds in patients with confirmed AHP. Because of potential thrombophlebitis from hemin, it is best given into a high-flow central vein via a peripherally inserted central catheter or central port. In addition, heme bound to human serum albumin is preferred due to heme stabilization and less irritation to veins.^34^

## Best Practice Advice 5: In addition to intravenous hemin, management of acute attacks of AHP should include pain control, antiemetics, management of systemic arterial hypertension, tachycardia, and hyponatremia and hypomagnesemia, if present.

The primary goal of treatment during an acute attack is to decrease ALA production. Identifiable precipitating factors, such as medications that induce cytochrome P450s, should be stopped, as they can directly up-regulate ALAS1 messenger RNA. Severe pain and nausea should be treated aggressively with analgesics and antiemetics. Intravenous carbohydrate loading (approximately 300 g/d in adults) is commonly used during the early stages of acute attacks, as studies have shown that fasting induces expression of peroxisome proliferator–activated receptor *γ* coactivator 1-*α*, which induces expression of ALAS1.^35^

Acute attacks can be associated with hyponatremia and hypomagnesemia due to a combination of hypovolemia and syndrome of inappropriate antidiuretic hormone secretion.^36,37^ Electrolytes should be monitored during acute attacks and hyponatremia corrected slowly if present.

In acute attacks that present with seizures, management should be approached with caution because many anticonvulsant medications, such as barbiturates, hydantoins, carbamazepine, and valproic acid, are contraindicated in AHP.^38^ Magnesium sulfate, benzodiazepines, and levetiracetam appear to be safe options in the context of AHP.

## Best Practice Advice 6: Patients should be counseled to avoid identifiable triggers that may precipitate acute attacks, such as alcohol and porphyrinogenic medications.

The low penetrance of AHPs indicates the likely presence of additional factors that are required for clinical symptoms, including precipitating factors and other poorly defined modifying genes and/or epigenetic factors.^39,40^ Sex hormones, in particular progesterone, are known to precipitate attacks. Other common precipitating factors include medications that induce cytochrome P450, acute illness or infection, physical or psychological stress, excess alcohol intake, tobacco use, and caloric deprivation. These factors induce hepatocyte ALAS1 messenger RNA and protein expression.^35,41–43^ All patients with AHP should be counseled to avoid identifiable triggers. Lists of safe and unsafe drugs are available online (https://www.porphyria.org/patient-resources/drug-safety-database-for-ahp/ and http://www.drugs-porphyria.org/).

## Best Practice Advice 7: Prophylactic heme therapy or givosiran, administered in an outpatient setting, should be considered in patients with recurrent attacks (4 or more per year).

Despite the improvement in management of acute attacks, treatment for patients with AHP with recurrent attacks remains challenging. Many patients are women who may experience menstrual cycle–associated attacks, which can be treated with hormonal suppression therapy, such as GnRH agonists, but with limited success.^44^

Off-label use of prophylactic intravenous hemin infusion is common.^1,45,46^ Although hemin is effective at stopping acute attacks, its effectiveness in preventing recurrent attacks is less established. In addition, chronic hemin use is associated with several complications, including the need for indwelling central venous catheters, infections, and iron overload. Patients receiving prophylactic hemin therapy should be screened for iron overload (see Best Practice Advice statement 9).

Recently, a novel small interfering RNA–based therapy targeting ALAS1 was approved by the US Food and Drug Administration and European Medicines Agency for treatment of adults with AHP (United States) or person 12 years and older (European Union).^47,48^ Givosiran is an ALAS1-specific small interfering RNA covalently linked to N-acetyl galactosamine that is administered subcutaneously and taken up selectively by hepatocytes via the asialoglycoprotein receptor.^49^ After uptake into hepatocytes, the small interfering RNA is processed by the cellular enzyme Dicer into approximately 20-bp-long single strands, which bind to ALAS1 messenger RNA and targets it for destruction, resulting in reduced translation of ALAS1 and decreased ALA production.^50^ In a phase 3, randomized, double-blinded, placebo-controlled study of patients with AHP with recurrent attacks, monthly subcutaneous givosiran significantly lowered rates of acute attacks that correlated with lower urine ALA and PBG levels.^47,51^ A significant proportion of patients on givosiran were free of attacks.^51,52^ We suggest prescribing givosiran only for those patients with recurrent acute attacks that are both biochemically and genetically confirmed. Due to limited safety data, givosiran should not be used in women who are pregnant or planning a pregnancy. Patients on givosiran should be screened and monitored for elevations in liver enzymes, serum blood urea nitrogen and creatinine, homocysteine, amylase, and lipase.^52^

## Best Practice Advice 8: Liver transplantation for AHP should be limited to patients with intractable symptoms and significantly decreased quality of life who are refractory to pharmacotherapy.

Liver transplantation has been successful in treating patients severely affected with AIP in reports from the United States and Europe.^42,53,54^ The primary rationale for the role of liver transplantation is that it corrects the HMBS deficiency in the liver of patients with AIP and restores normal PBG and ALA levels with resolution of clinical symptoms. Liver transplantation has been found to be curative in both the United States and Europe for patients with recurrent attacks refractory to treatment.^42,55^ Urinary ALA and PBG levels normalize after transplantation and some improvements in chronic symptoms, including peripheral neuropathy, have been reported.

In a recent report from Europe, retrospective data from the European Liver Transplant Registry were used to report on 38 liver transplant recipients (including 5 liver-kidney recipients) at centers across 12 countries in 2002–2019.^56^ Liver transplantations were deemed curative in all cases, except 1 patient who received an auxiliary transplant, and overall were associated with elimination of AIP attacks and significant improvement in neurologic complications. The 1-year and 5-year overall survival rates were 92% and 82%, respectively, with poorer outcomes in patients with severe neuropathy and/or renal dysfunction at baseline. As AHPs are predominantly autosomal dominant with low penetrance, carriers of pathogenic variants may be asymptomatic. If living donor transplantation is considered, genetic testing should be used to screen related living donors, as HMBS pathogenic variants in asymptomatic living donors may result in poor post-transplantation outcomes.^57^ Transplantation should ideally be performed in centers with experience with AHP, including preoperative management with hemin.

## Best Practice Advice 9: Patients with AHP should be monitored annually for liver disease.

Liver enzymes can be elevated in approximately 13% of patients with AHP during acute attacks and in some patients without clinical symptoms.^30,58^ In the ENVISION clinical trial of patients with recurrent AHP attacks, baseline serum aminotransferase elevations were reported in 28% of patients.^47^ Although chronic liver fibrosis and cirrhosis have been reported in patients with AHP, the cause of liver enzyme elevation and liver fibrosis is not well defined and, therefore, abnormal liver enzymes should prompt diagnostic investigation for alternative etiologies.^59^ In some cases, a liver biopsy may help diagnose concomitant conditions, such as nonalcoholic steatohepatitis, iron overload, or other causes.^60^

In addition, chronic hemin administration (9% iron by weight) can result in secondary iron overload with liver fibrosis. Liver enzymes should be monitored at least annually in AHP patients actively undergoing treatment. In patients receiving prophylactic hemin therapy at least monthly, ferritin and iron levels should be monitored every 3–6 months. In patients with ferritin levels >1000 ng/mL, therapeutic phlebotomy should be considered to reduce iron overload.^60^ Serum alanine aminotransferase elevation >3× the upper limit of normal has been reported in patients treated with givosiran during the first 3–6 months of treatment and, therefore, clinicians should monitor liver enzymes monthly during this period.^47^ If laboratory monitoring reveals normal findings, monitoring frequency may be decreased gradually to twice per year.

## Best Practice Advice 10: Patients with AHP, regardless of the severity of symptoms, should undergo surveillance for HCC, beginning at age 50 years, with liver ultrasound every 6 months.

Patients with AHP experience an increased risk of HCC (and cholangiocarcinoma), with risk estimates ranging from 1.5% to 1.8% in studies from the United States and Europe.^17,18,59,61–64^ Most cases of HCC have been reported in patients with AIP, whereas risk estimates in patients with VP and HCP forms are unknown. A meta-analysis of HCC in AHP confirmed a significant female predominance (2.5 times higher than in male patients), and several studies have revealed that HCC may occur in the absence of liver fibrosis or cirrhosis.^64^ Although HCC risk appears to be higher in clinically and biochemically active patients, it has also been reported in asymptomatic patients.^63^ Most studies identified HCC in patients with AHP who are older than 50 years. The role of *α*-fetoprotein in HCC surveillance in AHP is unclear, as *α*-fetoprotein elevation is not routinely observed in patients with high tumor burden. However, based on the estimated HCC risk of >1.5% in patients with AHP, HCC surveillance should begin at age 50 years with liver ultrasound every 6 months.^54,63^

## Best Practice Advice 11: Patients with AHP on treatment should undergo surveillance for chronic kidney disease annually with serum creatinine and estimated glomerular filtration rate.

The risk of chronic kidney diseas**e (**CKD) and hypertension is increased in patients with AIP. In the longitudinal study of the US Porphyrias Consortium, which enrolled symptomatic, asymptomatic, and latent carriers of AHP, CKD and hypertension were reported in 29% and 43% of patients, respectively.^19^ Systemic arterial hypertension is often seen during acute attacks and can result in chronic hypertension in some patients. In a report from France, porphyria-associated kidney disease (PAKD) occurred in up to 59% of the patients with symptomatic AIP, with an annual decline in the glomerular filtration rate of approximately 1 mL/min per 1.73 m^2^, and among patients with PAKD, approximately 60% had concomitant hypertension.^15^ PAKD is postulated to occur due to ALA and PBG-mediated endoplasmic reticulum stress, apoptosis, and epithelial phenotypic changes in proximal tubular cells leading to renal injury.^15,65^ In addition, it has been reported that a variant of the human peptide transporter 2 expressed by proximal tubular cells that mediate the reabsorption of ALA is an independent risk factor in developing PAKD.^66^

In the EXPLORE prospective multinational natural history study of patients with recurrent AHP attacks, 68% experienced reduced estimated glomerular filtration rate (eGFR) during the study,^10^ and 28% met the criteria for stage 3a, 3b, or 4 CKD, based on eGFR levels. Importantly, therapeutic interventions, such as givosiran, may be associated with worsening eGFR and renal function in a subset of patients based on ENVISION clinical trial and real-world data.^47,67^

Kidney transplantation is the treatment of choice for patients with end-stage renal disease secondary to AHP, as ALA and PBG levels can increase significantly between dialysis sessions.^14^ Immunosuppressive therapy post transplantation is generally well tolerated, and studies of kidney transplant recipients with AIP confirmed a significant improvement in clinical AIP symptoms, which is attributed to increased ALA and PBG clearance after transplantation.^68^

Based on these studies, serum creatinine and eGFR should be monitored at least annually in patients with AHP and more frequently in patients on givosiran.^60^ In addition, optimal control of systemic arterial hypertension is recommended to decrease additional risk factors for CKD.^15,60^

## Best Practice Advice 12: Patients should be counseled on the chronic and long-term complications of AHP, including neuropathy, CKD, hypertension, and HCC, and need for long-term monitoring.

The AHPs are chronic multisystem disorders that require ongoing monitoring and active management. Patients with recurrent attacks have a high chronic disease burden, including chronic nausea, fatigue, neuropathy, and kidney disease.^10,19,60^ In the EXPLORE natural history study, 65% of patients with recurrent attacks reported chronic symptoms, most commonly pain, fatigue, anxiety, and nausea, with 46% reporting daily symptoms.^10^ Quality of life is decreased considerably, with high rates of depression, anxiety, and insomnia.^10,12^ Although the disease burden in patients who are clinically asymptomatic with elevated levels of porphyrin precursors is not well defined, these patients may also experience an increased risk of CKD and HCC, and merit annual monitoring. Patients on chronic hemin or givosiran require close follow-up for adverse effects and treatment response.^60^

In conclusion, AHPs are diseases due to inherited partial defects in enzyme of porphyrin and heme synthesis. They are not as rare as previously thought. Fortunately, most people with genetic defects never experience severe acute attacks or may experience only one or a few attacks throughout their lives. Acute attacks, however, do occur, especially in women aged approximately 15–50 years, and some of these women experience monthly symptoms during the luteal phase of their menstrual cycles. The key to early diagnosis is to consider the diagnosis, especially in patients with recurring severe abdominal pain not ascribable to other causes. The key diagnostic test is a spot urine for ALA, PBG, porphyrins, and creatinine. Treatment of acute attacks should be with intravenous hemin given by peripherally inserted central catheter line or central port, 3–4 mg/kg body weight/d for 3–5 days. Givosiran should be considered in patients with biochemically and genetically documented AHP and with frequent attacks (4 or more per year). Long-term risks of AHPs include systemic arterial hypertension, chronic liver and renal diseases, and development of HCC, even in the absence of cirrhosis. Patients should have ongoing assessments for such potential complications.

## Figures and Tables

**Figure 1.**
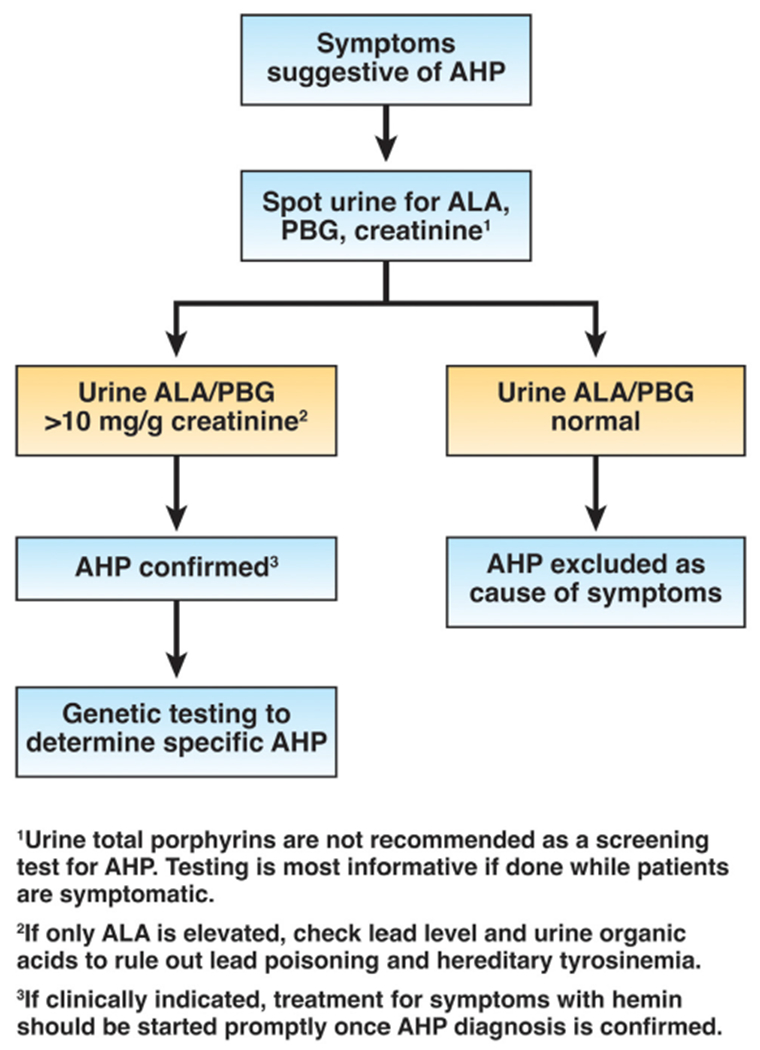
Diagnostic workflow for AHP.

**Table 1.** Summary of Acute Hepatic Porphyrias

AHP type	Inheritance	Age at presentation	Gender predominance	Symptoms and signs
AIP	AD	15–50 y	F > M	Acute
HCP	AD	15–50 y	F > M	Acute, cutaneous
VP	AD	15–50 y	F > M	Acute, cutaneous
ALAD	AR	Childhood-adulthood	M > F	Acute

AD, autosomal dominant; AR, autosomal recessive; F, female; M, male.

**Table 2.** Testing Recommendations for Acute Hepatic Porphyrias

Diagnostic biochemical testing	Confirmatory testing	Annual Monitoring	Monitoring on hemin^a^	Monitoring on givosiran^a,b^
Random urine PBG, ALA, and creatinine^c^	Genetic testing by sequencing *ALAD, HMBS, CPOX*, and *PPOX*	Liver enzymes, creatinine and eGFR, liver ultrasound, and *α*-fetoprotein every 6 mo after age 50 y	Iron, ferritin	Comprehensive metabolic panel, plasma homocysteine urinalysis; urinary protein to creatinine ratio, B12/folate, amylase/lipase

a Additional tests for patients receiving prophylactic hemin therapy or givosiran.

b We recommend that the listed tests be performed before the start of givosiran and again just before each monthly injection of givosiran for 3 months. If the laboratory test results are stable and the drug is being well-tolerated, we recommend that laboratory monitoring be repeated once every 3 months for the next year and at least once every 6 months thereafter.

c Sample should be normalized to creatinine, levels should be >5-fold the upper limit of normal for diagnosis. In 5-aminolevulinic acid dehydratase only ALA is elevated.

## Abbreviations used in this paper:

AHP: acute hepatic porphyria
AIP: acute intermittent porphyria
ALA: *δ*-aminolevulinic acid
ALAD: 5-aminolevulinic acid dehydratase deficiency porphyria
CKD: chronic kidney disease
eGFR: estimated glomerular filtration rate
HCC: hepatocellular carcinoma
HCP: hereditary coproporphyria
HMBS: hydroxymethylbilane synthase
PAKD: porphyria-associated kidney disease
PBG: porphobilinogen
VP: variegate porphyria
